# Open and Arthroscopic Surgical Anatomy of the Ankle

**DOI:** 10.1155/2013/182650

**Published:** 2013-10-29

**Authors:** Rachel M. Frank, Andrew R. Hsu, Christopher E. Gross, David M. Walton, Simon Lee

**Affiliations:** Division of Foot and Ankle Surgery, Department of Orthopaedic Surgery, Rush University Medical Center, Chicago, IL 60612, USA

## Abstract

Ankle-related complaints are among the most commonly encountered problems for musculoskeletal clinicians. Ankle pathology is widely variable, including, but not limited to, fractures, deformity, infection, oncologic diseases, neuromuscular conditions, and arthritis. While nonoperative management with activity modification, bracing and/or shoe modifications, and medications is usually indicated as first line of treatment, surgical intervention may become necessary. A thorough understanding of the complex anatomy and biomechanics of the ankle, and in particular, the potential neurovascular structures that may be encountered, is important to reduce complications and obtain good surgical outcomes. The purpose of this review is to discuss the most common open and arthroscopic exposures to the ankle with a focus on surgically relevant anatomy for each approach.

## 1. Introduction

Symptoms and complaints regarding the ankle are some of the most commonly encountered problems seen by musculoskeletal care providers. Ankle injuries encompass a broad array of pathology including trauma, deformity, reconstruction, and sports medicine. For nontraumatic injuries, physicians typically provide nonoperative treatment modalities to start including activity modification, rest, immobilization, bracing, orthotics, nonsteroidal anti-inflammatory medications, intra-articular injections, and physical therapy. When patient symptoms worsen and begin to negatively affect quality of living, surgical intervention often becomes necessary for definitive management. Patients with traumatic injuries, including fractures and/or dislocations, often require immediate surgical intervention. Regardless of the specific surgical technique performed, these procedures all require adequate visualization of the ankle pathology to be performed correctly.

A thorough understanding of the anatomy about the ankle joint, including the osseous, muscular, ligamentous, tendinous, and neurovascular structures, is critical to perform safe and effective ankle surgery. Open surgical exposures allow complete visualization of the tibiotalar articular surface and are the most commonly employed surgical approaches to the ankle. In recent years, less invasive ankle techniques including miniopen approaches and ankle arthroscopy have become more commonly used. The purpose of this review is to discuss the most common open and arthroscopic exposures used in the surgical treatment of ankle pathology with a focus on surgically relevant anatomy.

## 2. General Overview

**Figure 1:**
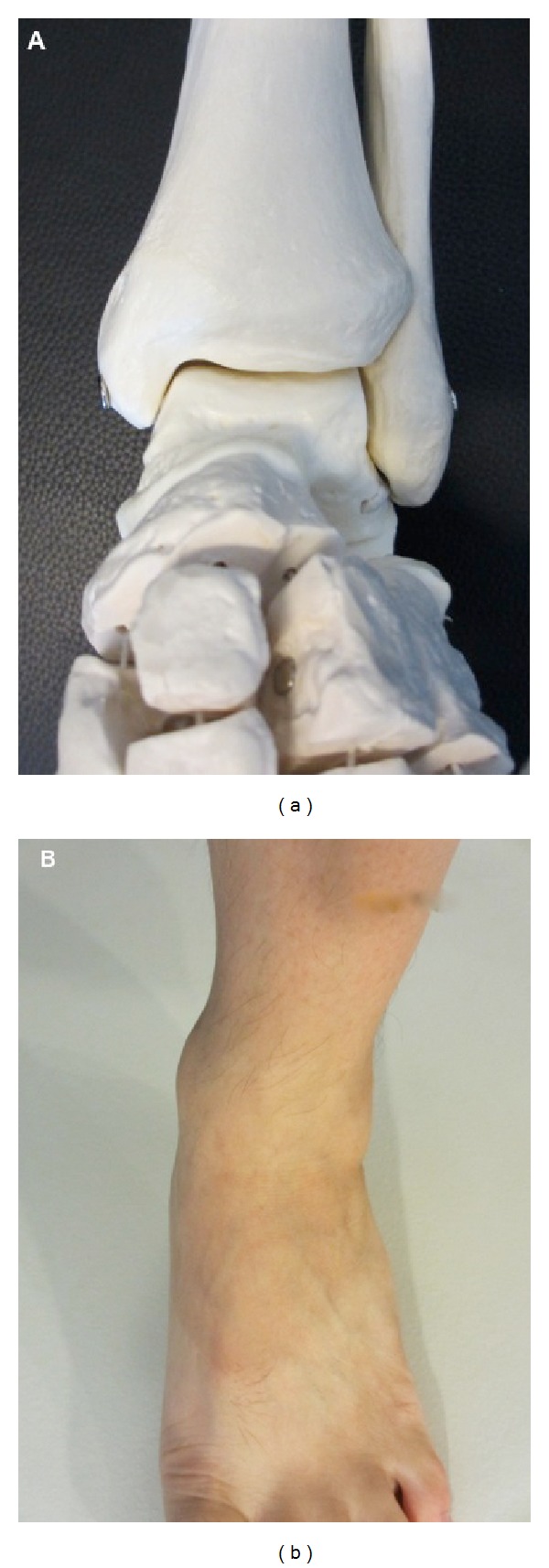
Superficial anatomy of the ankle as shown on a skeletal model (a) and patient (b); note the bone prominences of the medial and lateral malleoli and the level of the ankle joint.

**Figure 2:**
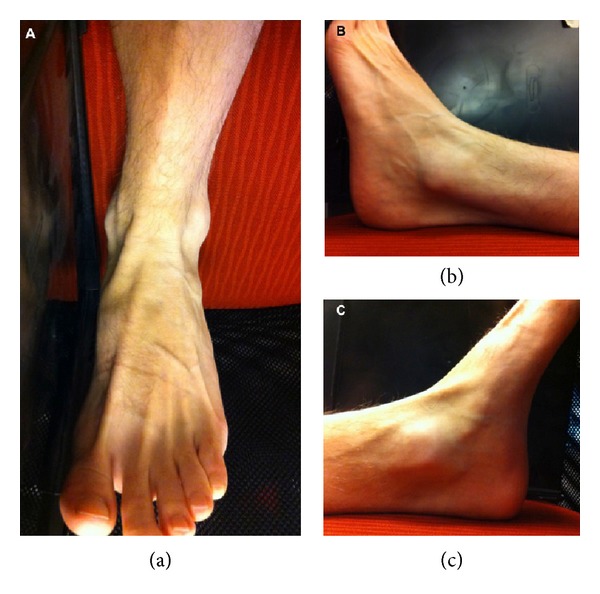
Topographical anatomy of the ankle joint as seen from anterior (a), lateral (b), and medial (c) views. Easily visible are the medial and lateral malleoli, the anterior tibialis tendon, and the tendons to the extensor digitorum longus.

The ankle joint is comprised of three bones including the tibia, fibula, and talus (Figures 1 and 2). The distal tibia forms an inferior quadrilateral surface that articulates with the talus and fibula to form a constrained joint. The fibula is externally rotated 25–30° relative to the distal tibia in the incisura fibularis, and the talus is wider anteriorly than posteriorly. Several soft tissue structures provide both static and dynamic stability of the ankle. These include the lateral ligamentous structures, medial ligamentous structures, syndesmosis, and the dynamic constraints provided by surrounding muscles and tendons.

### 2.1. Ligaments

The lateral ligamentous structures include the anterior talofibular ligament (ATFL), which resists anterior translation with the ankle in plantarflexion, talar tilt, and internal rotation, and the calcaneofibular ligament (CFL), which resists inversion of the ankle when in the neutral or the dorsiflexed position. The posterior talofibular ligament (PTFL) is the strongest of the lateral ligaments and plays a supplementary role in ankle stability when the lateral ligamentous complex is intact. The PTFL limits posterior talar displacement and external rotation and is under the greatest stress in dorsiflexion. The ATFL is the weakest of the lateral ligaments and extends from the anterior-inferior border of the fibula and inserts on the next of the talus. The PTFL originates on the posterior border of the fibula and inserts on the posterolateral tubercle of the talus. The CFL extends from the anterior border of the fibula to insert on the calcaneus, approximately 13 mm distal to the subtalar joint and deep to the peroneal tendon sheaths.

The syndesmosis consists of the anterior-inferior tibiofibular ligament (AITFL), posterior-inferior tibiofibular ligament (PITFL), transverse tibiofibular ligament, and interosseous ligament and membrane. The syndesmosis functions to maintain stability and integrity of the ankle mortise. Several specific anatomic features to the ankle joint are important to note when considering syndesmotic injuries and syndesmotic fixation. Specifically, the fibula is externally rotated 25–30° relative to the distal tibia in the incisura fibularis. During dorsiflexion, the fibula moves proximally and externally rotates to accommodate the wider anterior portion of the talus. While performing syndesmotic fixation, it is theoretically important to keep the ankle dorsiflexed while aiming the drill in a slightly posterior to anterior direction in order to maintain the normal anatomic relationship of the syndesmosis between the tibia and fibula.

The medial ligamentous complex of the ankle consists of the deltoid ligament. The deltoid ligament has two components (deep, superficial) and is the primary restraint to valgus tilting of the talus. Both layers resist eversion of the hindfoot and stabilize the ankle during plantarflexion, external rotation, and pronation. The deep portion of the deltoid ligament is the primary stabilizer of the medial ankle and resists lateral shift of the talus on the tibia; it originates from the posterior colliculus and inserts onto the medial and posteromedial aspects of talus. The superficial portion of the deltoid ligament resists subtalar eversion and external rotation of the talus; it originates from the anterior colliculus and inserts onto the navicular neck of the talus, sustentaculum tali, and posteromedial talar tubercle. The tibiocalcaneal portion of the superficial deltoid ligament is the strongest component of this layer and resists calcaneal eversion.

### 2.2. Muscles/Tendons

The peroneal brevis, longus, and tertius tendons course along the lateral aspect of the ankle, providing dynamic stability to the joint. The peroneus brevis inserts onto the base of the fifth metatarsal and functions to evert the foot. The peroneus longus inserts onto the base of the first metatarsal as well as the medial cuneiform and functions to plantarflex and evert the foot. At the level of the ankle joint, the peroneus longus is directly posterior to the peroneus brevis. The peroneus tertius inserts on the dorsal base of the fifth metatarsal and acts to dorsiflex, evert, and abduct the foot. It should be noted that the tibialis anterior (TA) is a direct functional antagonist of the peroneus longus as it inverts and dorsiflexes the ankle.

On the medial aspect of the ankle, several important structures, including the tibialis posterior, flexor digitorum longus (FDL), posterior tibial artery and vein, tibial nerve, and flexor hallucis longus (FHL), pass behind the medial malleolus from anterior to posterior. The posterior tibial tendon inserts on every tarsal and metatarsal bone except the first metatarsal via confluence with ligamentous structures. The FHL lies deep and dorsal to the FDL at the Knot of Henry and functions to flex the hallux. The specific anatomic locations of these tendons are critical to understand a variety of ankle pathologies. Not only do these tendons themselves often become irritated/inflamed/injured and require surgical intervention, but also they can become entrapped within and around the ankle joint in cases of trauma. For example, with lateral subtalar dislocations, the foot is locked in supination, and often it can be difficult to reduce the dislocation due to entrapment of the medial tendon structures (tibialis posterior, FDL, and FHL). Conversely with medial subtalar dislocation, the foot is locked in inversion, and barriers to reduction often include the peroneal tendons and/or the extensor digitorum brevis (EDB).

A full understanding of the complex anatomy of the ankle joint is necessary to effectively treat patients presenting with ankle pathology. A substantial number of structures critical to provide ankle joint stability exist within a relatively small area and in close proximity to neurovascular bundles. As will be discussed below, multiple vital vessels and nerves course throughout the lateral, medial, and anterior aspects of the ankle. Surgery is often aimed at fixing or correcting these anatomic structures (ligaments, tendons, and neurovascular structures) when they become injured or inflamed. Other times, surgery is not aimed directly at these structures (i.e., fracture, arthroplasty, etc.), and instead, they must be adequately identified, protected, and preserved throughout the entirety of the surgical case. Therefore, an appreciation of the intricate anatomy of the ankle joint is critical for safely and successfully performing surgical procedures. The subsequent sections will discuss the most common surgical approaches to the ankle with an emphasis on relevant surgical anatomy.

## 3. Open Surgical Approaches

### 3.1. Lateral

The lateral approach to the ankle is the common approach utilized in fracture surgery [1, 2]. This approach allows direct access to and complete visualization of the lateral malleolus, syndesmosis, and anterior and posterior aspects of the fibula. The lateral approach to the ankle is helpful in open reduction internal fixation (ORIF) procedures of the lateral malleolus, distal fibula, and syndesmosis. There is no internervous or intermuscular plane encountered with this approach. Landmarks used to help guide incision placement include palpation of the tip and body of the lateral malleolus as well as visualization of the short saphenous vein, which typically lies along the posterior border of the lateral malleolus.

**Figure 3:**
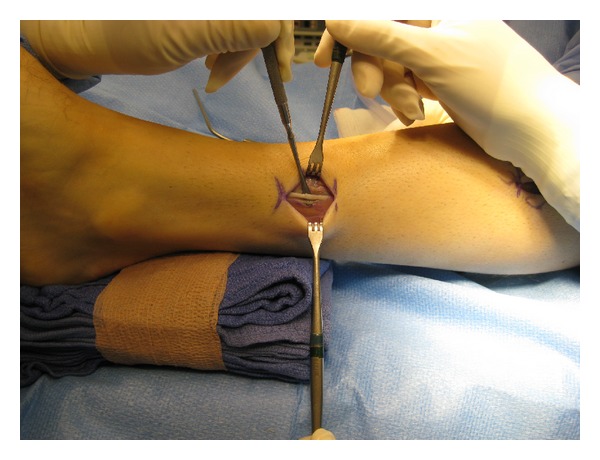
Intraoperative photograph demonstrating the location of the superficial peroneal nerve approximately 7–10 cm proximal to the lateral malleolus.

The incision is made in a linear fashion along the fibula centered over the fracture site in cases of fracture surgery. The incision can be extended distal to the tip of the lateral malleolus and proximal as well if an extensile exposure is needed. Dissection is continued superficially, taking care to create full-thickness skin flaps. Protection of the short saphenous vein and sural nerve is important, both of which are located posterior to the lateral malleolus. The superficial peroneal nerve (SPN) can commonly be found approximately 7–10 cm proximal to the lateral malleolus as it crosses over from the lateral to anterior compartments of the lower leg (Figure 3) [3]. If the SPN is encountered, care should be taken to protect the nerve and retract it anteriorly or posteriorly depending on the course of the nerve and exposure needed.

**Figure 4:**
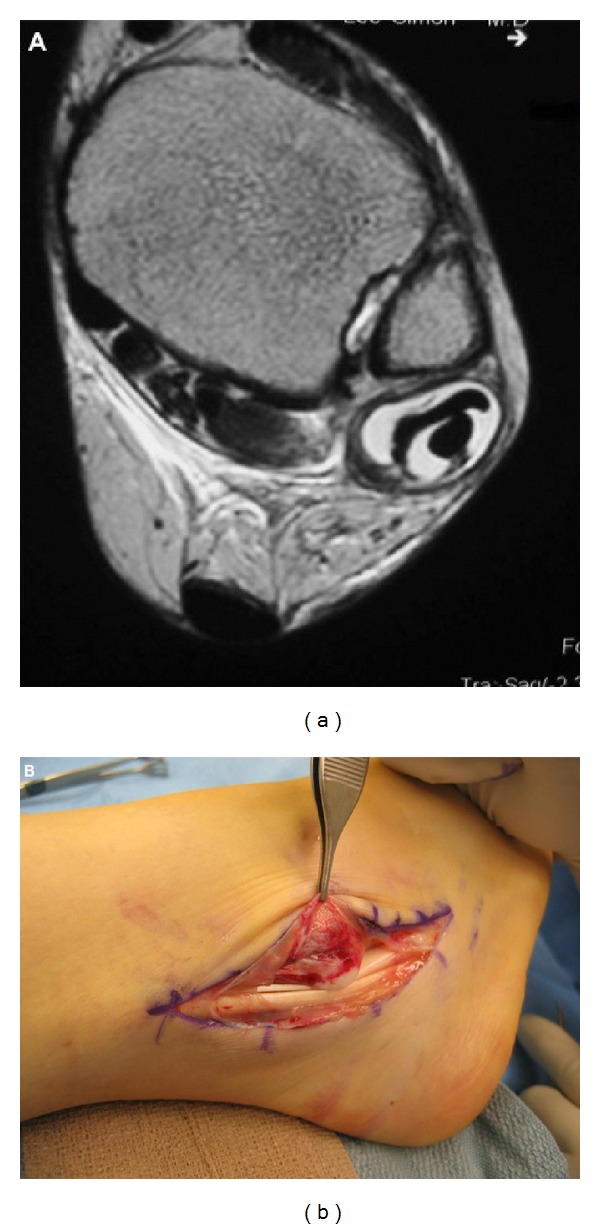
(a) T2-weight axial MRI image demonstrating peroneal tendon tendonitis (note the hyperintense (white) edema surrounding the hypointense (black) peroneal tendons); (b) intraoperative photograph demonstrating a lateral approach to the ankle and exposure of the peroneal longus and brevis tendons.

Deep dissection is continued in line with the skin incision through the periosteum overlying the lateral aspect of the fibula. Care should be taken to preserve as much of the periosteum as possible in order to allow uninterrupted blood supply to the bone; however, enough periosteum should be stripped in order to expose the fracture site. Following adequate exposure, the dissection can be continued anteriorly to visualize the syndesmosis. In addition to fracture surgery, the lateral approach to the ankle can be used and modified for treatment of peroneal tendon subluxation (Figure 4), lateral ankle instability, ankle arthrodesis, and other ankle pathologies. Through this same incision, if needed, the posterolateral tibia can be accessed between the peroneal tendons and the flexor halluces longus (FHL) as described in detail below.

#### 3.1.1. Structures at Risk

While this approach involves no true internervous plane, several neurovascular structures remain at risk. The structures include the sural nerve, the short saphenous vein, terminal branches of the peroneal artery, [4], and the SPN.

### 3.2. Posterolateral

The posterolateral approach to the ankle is also useful for ORIF procedures of the lateral malleolus and posterior malleolus [5–8]. This approach utilizes an internervous plane between the FHL, which is innervated by the tibial nerve, and the peroneal muscles, which are innervated by the SPN. For this approach, patients are usually placed in either the lateral decubitus or prone position, and landmarks include the calcaneus, Achilles tendon, and lateral malleolus. The incision is made in a linear fashion along the posterolateral border of the fibula. Dissection is continued superficially to the posterolateral edge of the fibula, taking care to create full-thickness skin flaps. The SPN may be visualized in the operative field approximately 7–10 cm proximal to the lateral malleolus and must be safely retracted. To gain full exposure of the distal fibula, retractors are used to posteriorly displace the peroneal muscles and tendons. To gain access to the posterior malleolus, the interval between the peroneals and FHL is entered. Retractors are used to anteriorly displace the peroneal muscles and tendons. Dissection is continued in this interval, and the FHL is elevated off of the posterior distal tibia followed by medial retraction. At this point, care must be taken to avoid devitalizing the posterior malleolus fragment and destabilizing the syndesmosis by inadvertently releasing the PITFL off of the distal posterior malleolus.

#### 3.2.1. Structures at Risk

With correct identification and utilization of the anatomical plane between the FHL and peroneal muscles utilized in the posterolateral approach, most neurovascular structures should be well protected. Specifically, the posterior tibial muscles and tibial nerve should be adequately protected behind FHL and retracted medially.

### 3.3. Medial

The medial approach to the ankle is a very common approach used in fracture surgery and osteochondral grafting of the talus [9–11]. This approach allows excellent access to and complete visualization of the medial malleolus, tibiotalar articular surface, and deltoid ligament. The medial approach to the ankle is helpful in ORIF procedures of the medial malleolus and can be modified to address injuries of the tibial plafond and deltoid ligament for repair and/or reconstruction. There is no internervous or intermuscular plane encountered with this approach. Landmarks used to help guide incision placement include palpation of the medial malleolus as well as visualization of the long saphenous vein.

**Figure 5:**
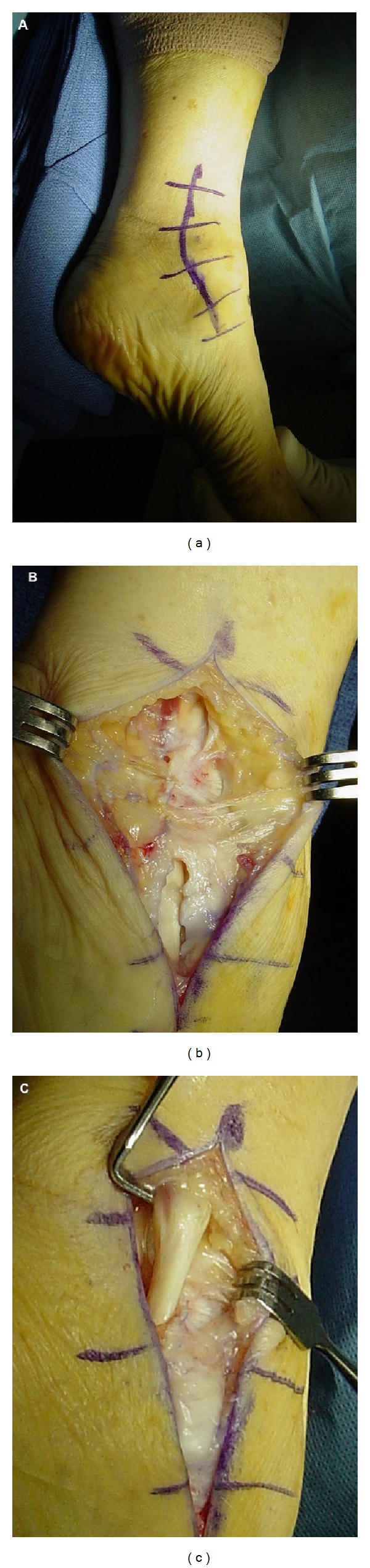
Intraoperative photographs demonstrating the (a) medial approach to the ankle, (b) superficial dissection, and (c) exposure of the posterior tibialis tendon for treatment of posterior tibialis tendonitis.

**Figure 6:**
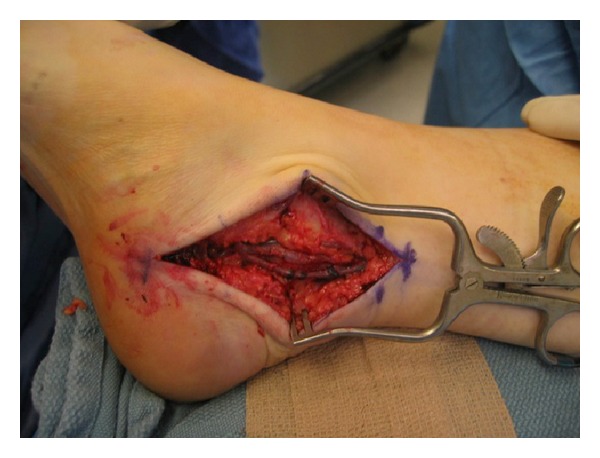
Intraoperative photograph demonstrating the medial approach and exposure of the flexor hallucis longus tendon for a patient with tarsal tunnel syndrome.

The incision is made directly over the medial malleolus, typically 7–10 cm in length in a curvilinear fashion with the posteriorly oriented apex of the curve at the medial malleolus. Superficial dissection is continued, and every attempt is made to create full-thickness skin flaps in order to aid with closure and prevent wound healing complications. During dissection, the long saphenous vein is usually found just anterior to the medial malleolus and should be preserved and retracted medially. Similarly, the long saphenous nerve will travel next to the vein and, if identified, should also be preserved; occasionally, the nerve is too small to be visualized. The dissection will continue directly to the medial malleolus periosteum at which point, in cases of fracture surgery, the periosteum can be elevated to better expose the fracture site. Through this approach, the deltoid ligament can be examined by extending the incision distally, and the anteromedial joint capsule can also be carefully incised in order to allow visualization of the articular surface of the tibiotalar joint. In addition to fracture surgery, the medial approach to the ankle can be used and modified for treatment of posterior tibialis tendonitis (Figure 5), tarsal tunnel syndrome (Figure 6), medial ankle instability, osteochondral lesions, and other medial ankle pathologies.

#### 3.3.1. Structures at Risk

The medial approach to the ankle is relatively safe with regard to avoiding injury to the neurovascular structures. However, the saphenous nerve and long saphenous vein typically run anterior to the medial malleolus and may block visualization during the surgical exposure. Both structures can usually be protected simultaneously if a thick mobile, anterior skin flap is created carefully during the superficial dissection.

### 3.4. Anterior

**Figure 7:**
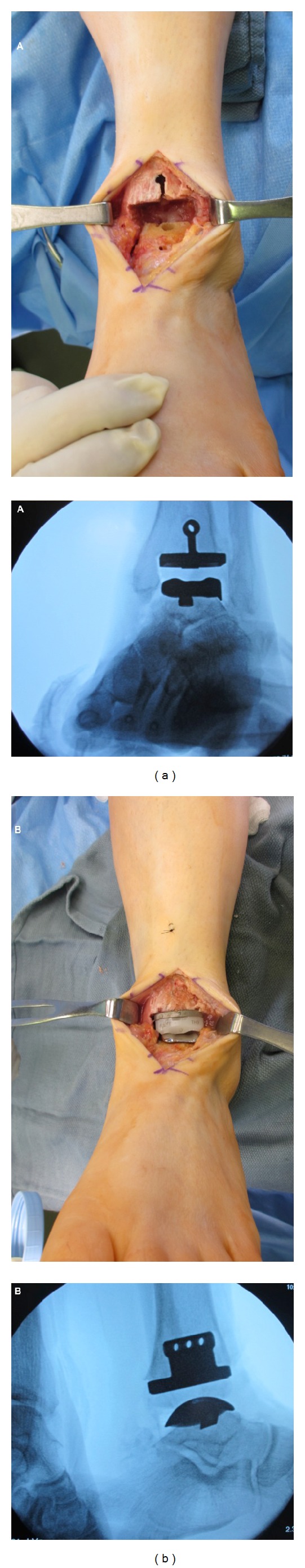
Intraoperative photographs demonstrating the anterior approach to the ankle with full exposure of the joint (a) and placement of components (b) in a patient undergoing total ankle arthroplasty; also, intraoperative fluoroscopic images are seen including an AP (a) and lateral (b), of the ankle with appropriate positioning of the hardware.

The anterior approach to the ankle is commonly employed for wide exposure of the distal tibia, tibiotalar joint, and talar dome [12, 13]. Common procedures utilizing this approach include total ankle arthroplasty (Figure 7), ankle arthrodesis, ORIF of pilon fractures [14], open irrigation and debridement of infections, and removal of intra-articular loose bodies. This approach utilizes an intermuscular plane between the extensor hallucis longus (EHL) and extensor digitorum longus (EDL), both of which are innervated by the deep peroneal nerve (DPN). Landmarks for this procedure include identification of the TA tendon, medial malleolus, lateral malleolus, and joint line.

The incision for this approach is made over the anterior ankle, beginning approximately 10 cm proximal to the joint line, and is extended distally in a linear fashion between the medial and lateral malleoli. The incision can be extended distally as needed to visualize the anterior talus and talonavicular joint. Initial dissection should remain superficial in order to avoid iatrogenic injury to the branches of the SPN that cross over the anterior aspect of the ankle from lateral to medial at this level. The dissection is continued, and the fascia is incised in line with the incision. Next, the extensor retinaculum is incised in line with the skin incision. The intermuscular interval between the EHL and EDL is identified 2-3 cm proximal to the joint line, and the EHL is retracted medially, while the EDL is retracted laterally. Of note, the anterior tibial artery and DPN travel in this area and should be directly identified, carefully protected, and retracted medially with the EHL. At this point, the anterior capsule of ankle joint is clearly exposed and can be incised in order to gain access to the joints and complete the intended procedure. Subperiosteal dissection medially and laterally can allow exposure of the entire ankle joint along with the gutters and inferior syndesmosis.

Anteromedial and anterolateral variations to the anterior approach have been well described and are often used for exposure of pilon fractures. The anteromedial approach [15] is similar to the anterior approach; however, the incision is made anterior to the medial malleolus, and after incising the deep fascia to the medial side of the TA tendon, the TA tendon is retracted laterally. The anteromedial aspect of the ankle has a small soft tissue envelope and is therefore more prone to wound complications after surgery. The anterolateral variation [16] involves an incision placed more laterally along the course of the peroneus tertius in line with the fourth ray. Following deep dissection of the fascia and extensor retinaculum, the anterior compartment tendons are elevated and retracted medially. This variation does place the SPN more at risk but has a larger soft tissue envelope for healing.

#### 3.4.1. Structures at Risk

The structures most at risk during the anterior approach to the ankle include the cutaneous branches of the SPN, which are at risk during the initial skin incision, as well as the DPN and anterior tibial artery, which are at risk during deeper dissection as they run between the EDL and EHL. Of note, this neurovascular bundle crosses behind the EHL at the level of the tibiotalar joint and must be protected at all times.

## 4. Arthroscopic Approach

Ankle arthroscopy has become a popular surgical approach for addressing many intra-articular ankle pathologies, including treatment of articular cartilage defects, removal of loose bodies, treatment of impingement, and repair of soft tissue injuries. Arthroscopic-assisted and all-arthroscopic procedures have also been recently described for tibiotalar arthrodesis and articular fracture reduction. A thorough understanding of both the superficial and deep anatomy of the ankle joint is critical for performing a successful and safe arthroscopic procedure without causing iatrogenic injury to the surrounding neurovascular structures [17–26]. Unlike in open surgery, where the majority of structures can be seen under direct visualization, in arthroscopy, the surgeon must know the exact locations of the structures at risk in order to avoid causing injury.

**Figure 8:**
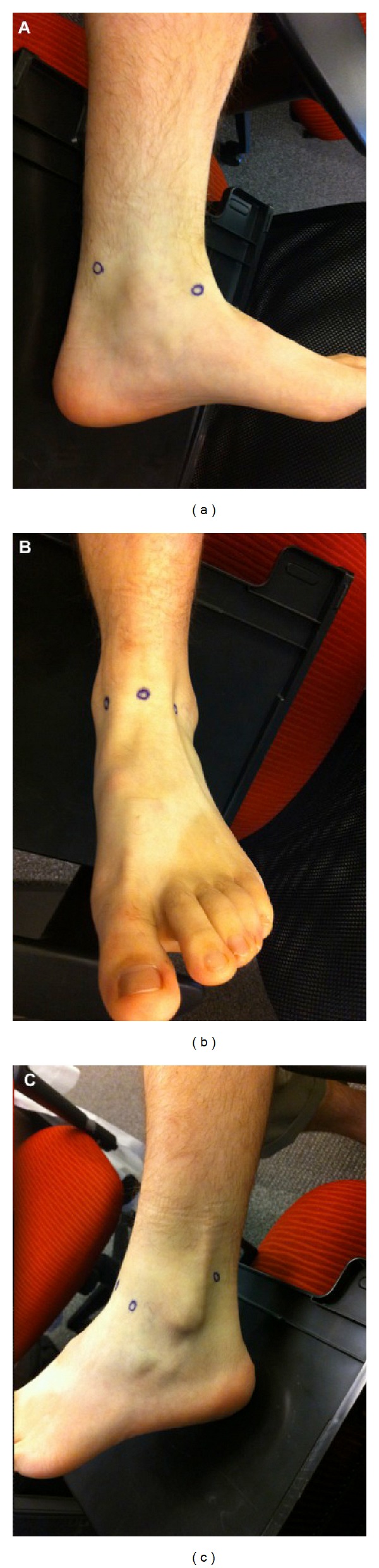
Intraoperative photographs demonstrating the appropriate position of ankle arthroscopy portals as seen from the (a) medial (PM and AM portals visible), (b) anterior (AM and AL portals visible), and (c) lateral (AL and PL portals visible) perspectives.

Pathology most commonly addressed with arthroscopy includes treatment of talar osteochondral defects, debridement of synovitis, and resection of impinging structures such as bony spurs, removal of loose bodies, and a variety of articular cartilage reparative and restoration procedures. Landmarks for ankle arthroscopy include palpation of the medial and lateral malleoli and palpation of the TA tendon and peroneal tendons. Several arthroscopic portals are utilized during ankle arthroscopy, including the anteromedial (AM), anterolateral (AL), posterolateral (PL), and posteromedial (PM) portals (Figure 8). The AM and AL portals are the two most commonly used portals for standard arthroscopic ankle procedures, including diagnostic arthroscopy.

**Figure 9:**
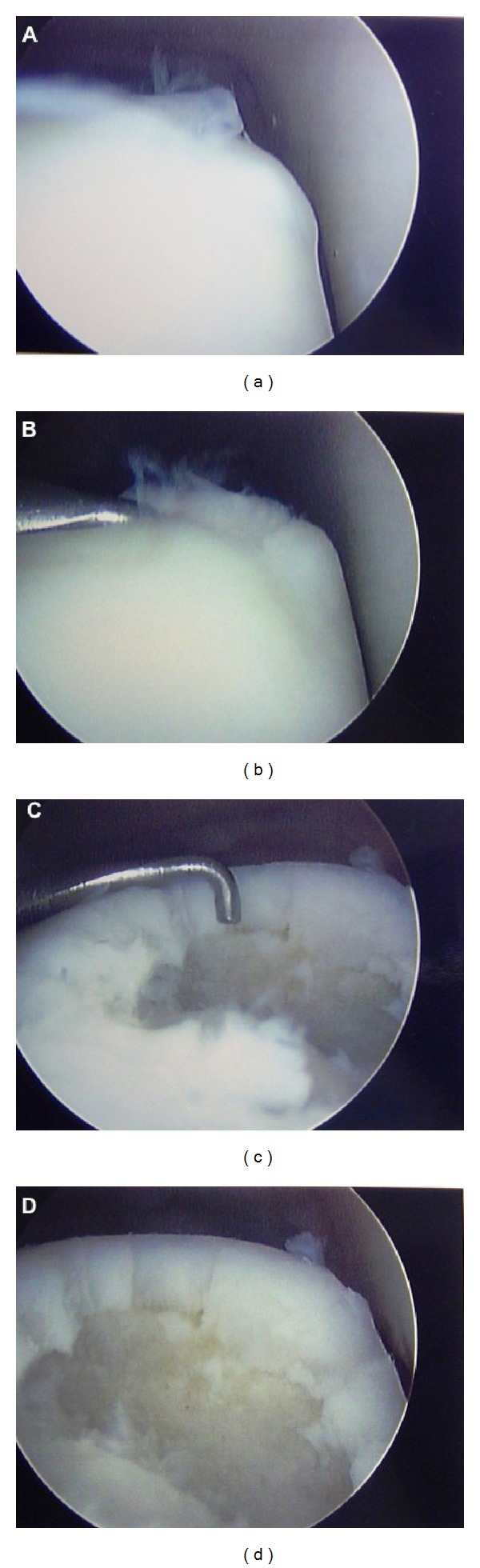
Intraoperative arthroscopic photographs demonstrating a medial talar osteochondral defect (a), probing of the unstable defect (b), and creation of stable vertical walls with use of an arthroscopic curette ((c),(d)).

The AM portal is the primary viewing portal and is established first after insufflation of the joint with an 18-gauge needle. This portal is established just medial to the TA tendon, typically between the TA tendon and the saphenous vein. Portals are made by incising the skin using a No. 11 blade scalpel. A hemostat is then used to bluntly dissect down to the capsule. A sharp trocar is then used to penetrate into the ankle joint. Once the AM portal is established and the arthroscope is inserted, the AL portal can be made under direct visualization. This portal is established just lateral to the peroneus tertius tendon, medial to the lateral malleolus. Care should be taken to make this portal lateral to the SPN as the nerve is approximately within 1-2 mm of the portal. One of the most common procedures performed utilizing the AM and AL portals is debridement of talar dome osteochondral defects [27] (Figure 9).

The posterior portals, including PL portal and PM portal, are not established when access to the posterior articular surface is needed as in cases of posterior osteochondral lesions, symptomatic os trigonum, and soft tissue impingement. The PL portal is established approximately 2 cm proximal to the tip of the lateral malleolus, medial to the peroneal tendons, and lateral to the Achilles tendon. In contrast, the PM portal is established at this level but just medial to the Achilles tendon.

### 4.1. Structures at Risk

A variety of neurovascular structures and tendons are at risk during ankle arthroscopy [28]. These same structures are at risk during open procedures; however, during open exposures, the structures are better visualized, and thus, it is easier to avoid iatrogenic injury. During establishment of the AL portal, the dorsal intermediate cutaneous branch of the SPN is at risk [29] and is the most common injury sustained during creation of this portal. As noted above, the saphenous nerve and greater saphenous vein are at risk during creation of the ML portal, the sural nerve and small saphenous vein can be injured during establishment of the PL portal, and the posterior tibial artery can be damaged during creation of the PM portal.

## 5. Conclusion

A variety of surgical approaches can be utilized in the treatment of ankle pathology. While the exposures are relatively straightforward and direct to the area of interest, a solid foundation of ankle anatomy is necessary to perform these procedures both safely and efficiently to avoid iatrogenic injury to the nearby structures. Damage to some of these structures, such as the SPN and the dorsalis pedis artery, can be devastating for the patient, leading to permanent morbidity and disability. By understanding the typical as well as sometimes variable anatomy about the ankle, regardless of the specific surgical approach chosen, both open and arthroscopic procedures required can be performed safely.
